# Current Approaches and Tools Used in Drug Development against Parkinson’s Disease

**DOI:** 10.3390/biom11060897

**Published:** 2021-06-16

**Authors:** Oliwia Koszła, Piotr Stępnicki, Agata Zięba, Angelika Grudzińska, Dariusz Matosiuk, Agnieszka A. Kaczor

**Affiliations:** 1 Department of Synthesis and Chemical Technology of Pharmaceutical Substances with Computer Modeling Laboratory, Faculty of Pharmacy, Medical University of Lublin, 4A Chodzki St., 20-093 Lublin, Poland; koszlaoliwia@gmail.com (O.K.); piotrstepnicki93@gmail.com (P.S.); agata.zieba@umlub.pl (A.Z.); grudzinska.ang@gmail.com (A.G.); dariusz.matosiuk@umlub.pl (D.M.); 2 School of Pharmacy, University of Eastern Finland, Yliopistonranta 1, P.O. Box 1627, FI-70211 Kuopio, Finland

**Keywords:** computer modeling, drugs, in vitro models, in vivo models, neurodegeneration, Parkinson’s disease

## Abstract

Parkinson’s disease is a progressive neurodegenerative disorder characterized by the death of nerve cells in the substantia nigra of the brain. The treatment options for this disease are very limited as currently the treatment is mainly symptomatic, and the available drugs are not able to completely stop the progression of the disease but only to slow it down. There is still a need to search for new compounds with the most optimal pharmacological profile that would stop the rapidly progressing disease. An increasing understanding of Parkinson’s pathogenesis and the discovery of new molecular targets pave the way to develop new therapeutic agents. The use and selection of appropriate cell and animal models that better reflect pathogenic changes in the brain is a key aspect of the research. In addition, computer-assisted drug design methods are a promising approach to developing effective compounds with potential therapeutic effects. In light of the above, in this review, we present current approaches for developing new drugs for Parkinson’s disease.

## 1. Introduction

Parkinson’s disease (PD) is currently the second most common neurodegenerative disease among the older population. It is predicted that by 2030, the number of sick people will increase to 9 million. The diagnosis is based on the finding of at least two symptoms from among: tremor, rigidity, bradykinesia and postural instability, and is further confirmed by performing a histopathological examination which indicates the presence of Lewy bodies—protein aggregates composed of alpha-synuclein—which is a useful biomarker for in vitro research. The pathogenesis of PD is multifactorial. The accumulated proteins cause degeneration of dopaminergic neurons, especially in the substantia nigra (SN) area, which reduces the concentration of dopamine and weakens the dopaminergic transmission, leading to the motor symptoms characteristic of PD. In addition to the SN area, loss of neurons is observed in the basal ganglia, hypothalamus and olfactory bulb. It subsequently triggers the transmission disorders in other systems, namely the cholinergic, glutaminergic, adenosine, GABAergic, serotonergic, noradrenergic and histaminergic systems. Neuron degeneration also occurs as a result of oxidative stress, caused by disturbances in calcium regulation and mitochondrial, lysosomal and proteasome dysfunction. Degeneration of other non-dopaminergic systems produces non-motor symptoms of PD, for which dopamine replacement therapy brings no therapeutic effect [1,2].

Due to the complexity of the disease, the etiology of PD is still not fully understood. It is found that environmental and genetic factors play an important role in its development. Age is the biggest risk factor: over 60 years of age, the risk of developing the disease fluctuates within 2%, while above 80 years, it increases to 4%. A higher proportion of cases is noticeable among men, and it is estimated that about 5% of cases are inherited. It has been studied that individual genes may be responsible for the disease. So far, 26 genes, which are referred to as PARK genes, have been associated with the disease, and their mutations have been shown to have a significant impact on the development of the disease. The reduction of parkin production or the production of mutant proteins leads to the accumulation of the toxic proteins and the degeneration of neurons. Mutations in parkin (PARK2), PINK1 (PARK6), DJ-1 (PARK7) and ATP13A2 (PARK9) have been shown to be responsible for the recessive form of adolescent PD, while mutations in SNCA (PARK1), which encodes the synuclein protein, and LRRK2 (PARK8) are responsible for the autosomal form of PD. Important mutations are also those related to the protein 35 (VPS35) and GBA1—the gene encoding β-glucocerebrosidase. These genes are involved in the processes that are usually disturbed in PD patients, such as mitochondrial metabolism, autophagy and proteostasis. Other risk factors related to the development of PD are mutations in the HLA-DQB1 gene and, above all, in the gene encoding the tau MAPT protein, the expression of which is used in various cell models [1].

In the search for antiparkinsonian agents, interest is focused on several molecular targets. A noteworthy one is the adenosine A2A receptor. It has been investigated that the inhibition of this receptor increases the level of dopamine and improves signaling transmission. In animal models, blockade of A2A receptors has been proven to alleviate motor symptoms of the disease. It is likely to have neuroprotective effects as well [3,4].

An interesting molecular target is also monoamine oxidase B. In clinical studies, it has been shown that inhibition of these enzymes increases the level of dopamine in the brain by preventing its degradation. This has a positive effect on the motor and non-motor symptoms of PD, especially in the early stages of the disease [5].

Groundbreaking research focused on the polypyrimidine tract-binding (PTB) protein, which is responsible for turning genes on and off in the cell. In animal models, blockade of PTB has been shown to lead to the conversion of astrocytes into dopaminergic neurons, which restores normal dopamine transmission. This gives hope for an effective treatment that can cure PD permanently [6].

Standard treatment of PD focuses on increasing the levels of dopamine in the brain and preventing it from breaking down. However, this treatment does not inhibit the progression of the disease. The main problem in the treatment is that the exact etiology of the disease is not known, and it is not known what exactly initiates the neuronal damage process. There is still no drug that would effectively cure the disease. However, scientists are still trying to develop more effective pharmacotherapy by using new molecular targets based on relevant in vitro and in vivo models, and by using computer-aided design of drugs and other particles.

## 2. In Vitro Models for Parkinson’s Disease Studies

Various cellular models are required to develop new therapeutic strategies and to thoroughly investigate the pathomechanism of PD. First of all, the key point in selecting the appropriate model is to consider the aim of the experiment and the limitations of the model systems. The choice of line also depends on the aspect of the disease and the type of therapy we want to develop. Cell cultures are a very good model for research because they are less expensive than in vivo tests and develop pathology faster, and genetic and pharmacological manipulations are relatively easy and reliable [7].

Currently, PD research is conducted mostly in established cell models. Such models include the neuroblastoma line (SH-SY5Y). It is the line of choice for PD research due to its human origin, catecholaminergic and neuronal properties. Moreover, the line is very easy to maintain and conducts cell culture [8]. SH-SY5Y cells are a subtype of the SK-N-SH line that was obtained from a bone marrow biopsy of neuroblastoma. SK-N-SH was subcloned three times. First to SH-SY, then to SH-SY5, and finally, to SH-SY5Y [9]. Neuroblastoma cells can be differentiated into mature human neurons with the help of neurotrophins (brain-derived factor—BDNF) or retinoic acid [10]. Moreover, literature data show that differentiated SH-SY5Y cells are characterized by the presence of markers typical of mature neurons. The neuron biomarkers present in SH-SY5Y include: NeuN protein, which is a neuronal nuclear antigen, synpatophysin protein (SYN), neuron-specific enolase (NSE) or a protein related to the growth and plasticity of neurons (GAP-43) [11]. The use of various methods of cell differentiation also allows the selection of specific subtypes of neurons (adrenergic, cholinergic, dopaminergic neurons). SH-SY5Y cells also express dopamine β-hydroxylase or secretion norepinephrine [8,12], and therefore they can increase catecholaminergic activity. Moreover, the SH-SY5Y cell line is a good model for the study of disturbed dopamine homeostasis, which is an important aspect in the development of PD. The production of free radicals caused by the spontaneous oxidation of dopamine causes the malfunction of the mitochondria, and consequently increases oxidative stress, which in turn plays a key role in the development of neurodegenerative disorders [13]. The presented line, due to its neoplastic origin, is also characterized by many genetic aberrations, however, most of the genes and pathways involved in the pathogenesis of PD remain unchanged, and therefore it is one of the most frequently chosen models for research [8].

The next cell model most commonly used for PD studies is the PC12 line, derived from a pheochromocytoma of the rat adrenal medulla. PC12 cells are an excellent model for studying neurotoxicity in PD due to their unique properties that help to reflect pathogenic mechanisms. First of all, it is very easy to induce mitochondrial dysfunction in cells, which accompanies the pathogenesis of the disease with the use of 6-hydroxydopamine or rotenone [14]. Moreover, PC12 are directed to different cell death mechanisms as a result of overexpression and deposition of synuclein, which plays a key role in PD development and is one of the biomarkers of the disease [15]. As with SH-SY5Y cells, the PC12 line is capable of synthesizing and releasing catecholamines, which are thought to be further potential biomarkers in PD [16]. The differentiation of phaeochromocytoma is mediated by the action of the neural growth factor (NGF). NGF-mediated stimulation leads to an increase in the volume of cells, changes in their shape and to the generation of neuritic processes also by promoting the growth of axons. In response to the nerve factor, cells exhibit the properties of sympathetic neurons, and their differentiation occurs through the TrkA receptor [17,18,19]. The presented model of the PC12 line is a simple and commonly used in vitro model for studying the pathomechanism and modern methods of treating PD. However, as in the case of SH-SY5Y cells, it should be remembered that this is a model of cancer origin and has its limitations [14].

It is also worth mentioning that fibroblasts are good models for PD studies. Fibroblasts from PD patients can be successfully differentiated into dopaminergic neurons [20]. Available stem cell research reports that fibroblasts can be reprogrammed to a pluripotent state and then generate induced pluripotent stem cells (iPSC) [21]. iPSC-based cell models are very useful tools for studying the molecular mechanisms of PD [22].

Another advanced in vitro model for PD research is the LUHMES line. It is an immortalized human fetal mesencephalon cell line. These cells are a subclone of the MESC2 line [23]. LUHMES cells are maintained in an undifferentiated stage of proliferation by introducing the v-myc tetracycline-responsive (TET-off) gene. This model can be differentiated using tetracycline, glial cell line-derived neurotrophic factor (GDNF) and cyclic AMP (cAMP). As a result of the application of such factors, cells exit the cell cycle, thanks to which they successively differentiate into post-mitotic neurons [24]. After differentiation, neurons show phenotypically similar features to human dopaminergic neurons, and also have biochemical expression of markers characteristic of mature neurons. LUHMES exhibit the same neuronal and dopaminergic properties as the parent MESC2 cells. Moreover, LUHMES cells primarily show high expression of the L1CAM and α-synuclein, which are useful markers for studying PD. Moreover, the expression of the microtubule-associated protein tau (MAPT) and synapsin-1 (SYN1) proteins occurring in the presented cellular model is an important factor responsible for the risk of PD and the basic pathomechanism of this disease [24,25]. The presented cell model is very suitable for PD studies due to its human origin. Compared to the models presented above, the LUHMES line has no limitations due to its origin and may be better referenced in the in vivo testing system.

In recent years, three-dimensional (3D) cell cultures have been one of the most desirable and modern in vitro models for the study of neurodegenerative diseases. Organoids are made up of stem cells or progenitor cells. Organoid culture is based on the use of a special extracellular matrix and appropriate culture media that reflect the physiological environment. Various types of matrix are used to provide structural support, reflect the extracellular matrix and support 3D cultures so that cells retain their features and functions. The most commonly used matrix for obtaining organoids is Matrigel [26,27]. It contains a mixture of proteins secreted from mouse sarcoma cells EHS and collagen, laminin or proteoglycan, as well as various growth factors. Differentiated stem cells show the ability to self-organize into three-dimensional structures, while expressing proteins and nucleic acids [28]. The created 3D environment in which cells are grown is the closest to the conditions in a living organism. Moreover, the 3D cells exhibit a similar physiological response compared to human cells, which cannot be achieved with traditional two-dimensional cultures (2D). Innovative technology of spatial culture allows for the development of brain organoids, which, by ensuring interactions between nerve cells, have extensive potential to use them as a precise cell model for the study of PD. The development of many different 3D cultures allows not only the discovery of complex biological mechanisms and processes, but also creates the opportunity to generate potential compounds related to numerous diseases, including PD [29].

Diversity in the selection of appropriate cell models provides scientists with many options in the development of modern medicinal substances and understanding the pathomechanism of diseases.

## 3. In Vivo Models for Parkinson’s Disease Studies

In addition to the in vitro models mentioned above, research into PD using an appropriate animal model is invaluable. Constructive models should have characteristics of human PD and treatment effects using the models should reflect clinical treatment. For many years, various animal models have been developed to reflect the pathology of PD [30].

The most classic and most used in vivo models are neurotoxin models. They are very easily obtained by administering toxins that cause neuronal degeneration. One such compound is 6-hydroxydopamine (6-OHDA). 6-OHDA is used to generate a PD model by damaging the nigrostriatal dopaminergic system [31]. Moreover, this model is credible due to the presence of 6-OHDA in the human brain [31,32]. Oxidopamine is used both in in vitro and in vivo research. According to the available literature data, 6-OHDA may cause the degeneration of dopaminergic and noradrenergic neurons. Both types of neurons are highly susceptible to the toxic effects of 6-OHDA, because the dopamine and noradrenaline transporter has a strong affinity for this molecule [30]. The main cause of neuronal death is disturbed mitochondrial function as a result of the resulting oxidative stress associated with high levels of free radicals [33]. Depending on the amount and site of injection of the neurotoxin, it causes a different size and characteristic of neurodegeneration. In developing an animal model of PD, unilateral injection of 6-OHDA into the substantia nigra, medial forebrain bundle or striatum is most commonly used (hemi-parkinsonian model). Injecting of the toxin into one hemisphere allows the other side to be left intact, which can be used as an internal control for each animal. The presence of a damaged and intact hemisphere is particularly useful in behavioral analysis of PD. Bilateral injection of 6-OHDA is highly invasive and can cause aphagia and death in animals. According to the available literature data, the neurotoxic animal model using 6-OHDA is very widely used in research on PD, however, this model does not fully reflect the pathological features of PD, but only displays the symptoms of the disease [30,31].

Another compound used in the creation of animal models from the group of neurotoxins is 1-methyl-4-phenyl-1,2,3,6-tetrahydropyridine (MPTP). The mechanism of MPTP toxicity occurring in neurons has been thoroughly investigated. The accumulation of toxins in dopaminergic neurons induce neurotoxicity through disturbed mitochondrial function and an increase in reactive oxygen species [34]. The routes of administration of the toxin vary according to the species of the model. The most common and repeatable method is subcutaneous or intravenous injection, which shows bilateral parkinsonian syndrome. Another, equally frequently used method of administration is unilateral injection into the carotid artery, primarily inducing unilateral parkinsonism [34,35]. Animal models that use MPTP include primate species as well as sheep, guinea pigs, dogs and cats. Depending on the strain, mice show differences in sensitivity to MPTP. Moreover, for unknown reasons, rats are resistant to this toxin. In monkey and mouse models, administration of toxic MPTP causes damage to the nigrostriatal dopaminergic pathway. Moreover, in the mouse model, inclusions of ubiquitin, α-synuclein, can additionally be detected, and also at the locus coeruleus, Lewy bodies are present, and degeneration of noradrenergic neurons takes place. MPTP is most commonly used to build toxin-based models of PD [31,35,36].

There are also animal models where different types of pesticides/herbicides are used during development. Paraquat is used to generate such a PD model. It is an herbicide that has a similar molecular structure to MPTP. This compound causes toxicity, inter-alia, by causing oxidative stress as a result of the generation of reactive oxygen species. Administration of paraquat to mice resulted in the loss of midbrain dopaminergic neurons, striatal cells and reduced motor functions. An important aspect of the use of paraquat is its ability to induce α-synuclein expression and create Lewy body-like inclusions. The animal model using paraquat may be a valuable clue for research into the treatment of PD [31,35]. Rotenone is a pesticide also used to produce in vivo PD models. The intraperitoneal administration of rotenone causes neurodegeneration and a behavioral deficit. When administered intravenously, dopaminergic neurons are damaged by aggregation of α-synuclein and Lewy body-like inclusions. Intravenous administration also generates oxidative stress. The presented model shows promising results to be used to reflect PD pathology, however, it still requires a detailed study [35]. There are many different animal models for studying PD based on a variety of compounds (toxins) (e.g., isoquinoline, α-methyl-*p*-tyrosine, reserpine), however they have many limitations, and they are not scientifically valid models for research as the models presented above.

Neurotoxin models are continually used in PD research, but technological advances have led to the creation of genetic models. Such methods are a relatively new approach based on the genetic manipulation of animals. Animal models with mutations in the genes that occur in PD can recreate the pathophysiology of the disease and represent potential therapeutic targets [35,36]. In addition, the development of models with the appropriate mutation allows for a detailed study of the molecular and biochemical pathways in PD. α -synuclein mutations are one of the molecular targets genetically linked to PD. Two mutations in the α-synuclein gene were used to create an animal model of PD: A53T, A30P. The A53T mutation in mice showed severe motor dysfunction and inclusions similar to Lewy bodies. In addition, studies with different types of α-synuclein transgenic mice did not show significant neurodegeneration of dopaminergic neurons. High synuclein levels are associated with strong PD progression, and therefore PD models are based on this phenomenon [30,35]. Another genetic model is related to the LRRK2 mutation. Expression of mutant LRRK2 leads to neurochemical and behavioral pathology. In the proposed model, there is a decrease in motor activity and axonopathy of dopaminergic neurons with the presence of hyperphosphorylated tau protein. The model also provides a detailed analysis of the molecular mechanisms involved in neurodegeneration resulting from the LRRK2 mutation [37,38]. In addition, mutations in the Parkin gene are also used to create animal PD models. Parkin’s malfunction causes mitochondrial dysfunction and the accumulation of abnormal proteins. In vivo knockout models of this gene are a promising model for PD studies, and its regulation is a fundamental therapeutic strategy. The presented transgenic models enable the reproduction of many aspects of pathogenic PD, however, they require additional modification and clarification [30,39].

Overall, as a result of continuous research and technological development, various animal models for PD research have been developed. Although the animal studies carried out reflect the pathology of PD, such models may show great variability in the development of neuropathology depending on the injection site and type of substance, as well as the strains and animal species used.

## 4. Computational Approaches Used in the Development of Novel Drugs against Parkinson’s Disease

Despite the extensive research carried out on PD itself, the cascade of reactions that leads to the formation of this condition remains unknown. Moreover, PD is currently considered an untreatable disease, and advanced research provides novel theories that can contribute to designing more accurate medication [40].

**Figure 1:**
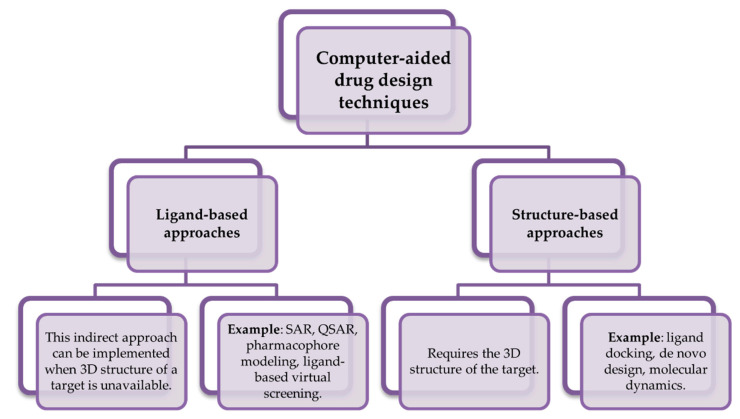
Classification of computer-aided drug design techniques (CADD) and the most commonly used methods. SAR—Structure-activity relationship; QSAR—Quantitative structure-activity relationship.

At first, medicinal chemistry programs focused on developing compounds that possessed a high selectivity against one type of molecular target. This approach was also known as “one-disease-one-target” [41]. In silico methods use computer-based approaches to accelerate the lead hit identification and molecule optimization. These methods are widely known as computer-aided drug design techniques (CADD) and can be divided into two categories: ligand-based and structure-based approaches (Figure 1). The former can be implemented when the structure of the protein target is not known. However, if there is some data on the molecular target structure, the latter methods can be used. These techniques enable identifying common regions in a series of active and inactive molecules [42,43]. Some of these techniques, e.g., molecular docking, pharmacophore identification, structure-activity relationship (SAR), quantitative structure-activity relationship (QSAR) and combination methods, have been widely applied in the process of designing novel, selective ligands [44].

So far, several reviews have been published that gathered information about the advances in the development of selective ligands [45,46,47,48,49,50,51]. Hamilton et al. published a complex review that collects information about the rational drug design studies performed using QSAR, molecular docking, molecular dynamics and pharmacophore modeling, that were applied in the development of drugs against PD [52]. This paper puts a special emphasis on the widely recognized molecular targets—dopaminergic receptors, acetylcholine receptors, monoamine oxidase and adenosine receptors. Such selective medication could be administered as a drug cocktail, combining several medicines or multi-component drugs (collecting several active ingredients in one formulation) [53]. Unfortunately, polypharmacy has many drawbacks. Thus, it has not met the high expectations in symptom mitigation, disease modification, and did not cure the disease itself. It turned out that the old concept of providing patients with various products containing only selective ligands no longer fulfilled the requirements of an effective pharmacotherapy [54].

Growing evidence supporting the theory that neurodegenerative diseases have complex mechanisms brought a new light into their pharmacotherapy and increased interest in the field of developing multi-target ligands [55,56,57]. In this context, a proper medication should acquire the criteria of many pharmacophores [58]. Various terms were used to describe such agents, e.g., dual ligands, heterodimers and pan agonists [59]. The multi-target theory led to the formation of novel drug design strategies, stating that medication should act at different levels of the pathomechanism in various targets. Therefore, to develop such pluripotential medicines, it is essential to examine multiple groups of compounds working through various mechanisms used to treat certain diseases. Collected information about structural features responsible for the proper interaction with drug target was used to form molecules with desired selectivity profiles. Many methodologies can be used to form such multi-target ligands. However, they can be classified either in one of these two main concepts: knowledge-based and screening approaches. The first technique takes advantage of currently available data on old drugs or historical compounds. It usually involves a selective combination (via appropriate linkages) of pharmacophores obtained from distinct medication groups. This enables the incorporation of favorable biological properties of these selective ligands.

On the other hand, such process may only require the incorporation of structural features responsible for the biological activity of a specific molecule into the structure of a compound activated by other drugs. The most commonly used approach requires compounds that possess a minimal activity against both desired molecular targets [59]. In contrast, the screening concept involves screening diverse or focused libraries of compounds that enable finding ligands which simultaneously interact with two desired targets [44,59].

These concepts significantly impacted the treatment of neurodegenerative diseases such as PD. Interestingly, recently, the screening approach has been widely used in the development of multi-target ligands useful in the treatment of neurodegenerative disorders [60,61,62,63,64,65,66]. Jaiteh et al. performed molecular docking screens against the binding sites of A_2A_ adenosine receptor and monoamine oxidase B (MAO-B) to identify dual inhibitors of these targets. The set consisting of 24 most promising docking results was evaluated experimentally, and that led to the identification of four dual-target ligands. Authors claimed that compounds possessing this dual activity would have the neuroprotective effects of adenosine receptor antagonists combined with the dopaminergic regulatory effect of MAO-B inhibitors. Zhi-Dong et al. performed a docking-based virtual screening to identify ligands that would interact with TP53, CASP1 and HSP90AB1 proteins involved in the pathomechanism of PD. Virtual screening hits were evaluated with a novel artificial intelligence protocol and used in molecular dynamics studies. This study revealed three potential candidates with desired properties, and according to the authors, these compounds will be submitted for a more detailed evaluation [67].

Artificial intelligence (AI) techniques, especially machine learning, are widely applied in many areas of life sciences. These methods are considered to possess the potential to assist the pharmaceutical industry and accelerate drug research by extracting novel and essential therapeutic information from large volumes of data [68,69]. Moreover, these approaches are believed to be a powerful tool in predicting new drug targets of gene–disease relationships [70]. Even though these techniques are relatively new, their application delivered some promising results in the field of drug design, e.g., Shao et al. used two AI methods—support vector machine models (SVM) and Tanimoto similarity-based clustering analysis—to identify ligands targeting adenosine A_2A_ and dopamine D_2_ receptors that contain indole-piperazine-pyrimidine moiety. Moreover, an excellent review article has been published by Vatansever et al. that gathers information about the main AI algorithms and their application in different stages in the development of medication against the central nervous system diseases [68].

In the end, it is worth emphasizing the contribution of network pharmacology (NP), a technique that changed the drug design strategy from “one-disease-one-target” to a multi-target ligand approach [71]. The network pharmacology approach uses techniques derived from computer science, molecular biology, pharmacology, etc. This concept focuses on analyzing large biological datasets and deriving links between compounds, specific proteins/genes and diseases [72]. This concept has been applied in several medicinal chemistry studies [72,73,74,75] and led to the identification of diverse novel targets and potential ligands.

In light of all these findings, extensive technological growth accompanied by continuous research on PD seems to be essential for the development of more efficient drugs.

## 5. Recent Reports of Novel Agents against Parkinson’s Disease

Current treatment of PD relies on alleviating its symptoms via regulation of dopaminergic neurotransmission. Efforts are being made to develop therapies that would address the causes and slow down the progress of the disease, however with very limited success to date. Another popular approach consists in designing multi-target molecules that are able to simultaneously affect several targets of interest. Here, we present reports of promising novel compounds with potential application in PD, that were designed and studied in the last five years.

**Figure 2:**
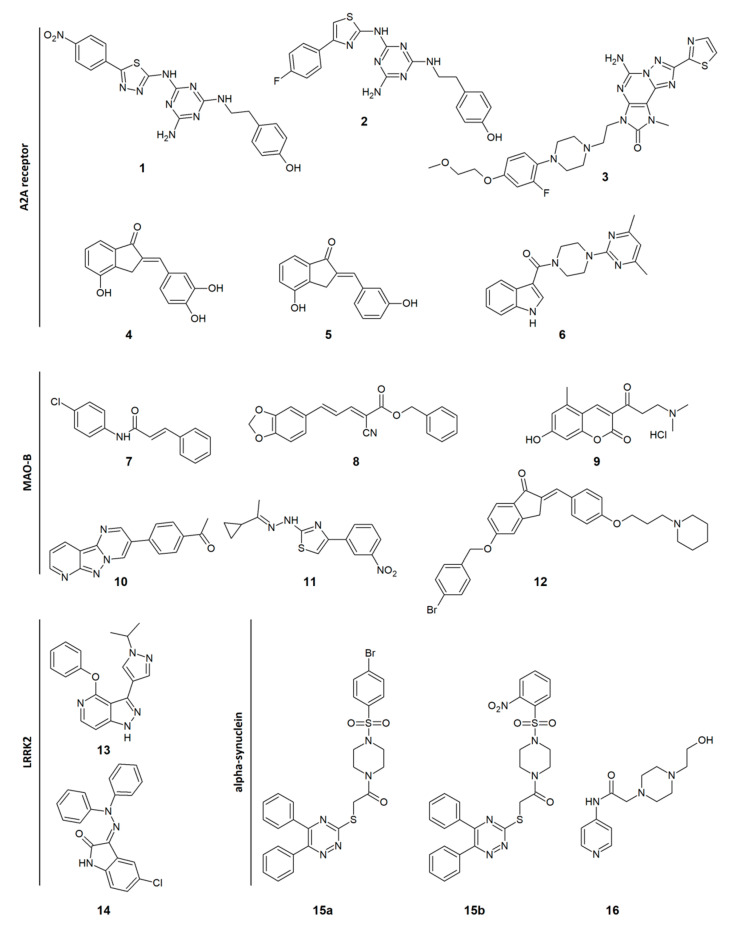
Chemical formulas of exemplary novel agents for potential application in the treatment of Parkinson’s disease. A2A—Adenosine A_2A_; MAO-B—Monoamine oxidase B; LRRK2—Leucine-rich repeat kinase 2.

One of the molecular targets in PD that recently drew scientists’ attention is adenosine A_2A_ receptor. Its inhibition in striatum leads to enhanced dopamine transmission, which may be beneficial for managing the symptoms of the disease. Masih et al. reported a series of novel 1,3,5-triazine-thiadiazole derivatives with affinity for adenosine A_2A_ receptor. In particular, compound **7e** (**1** in Figure 2) showed high potency and selectivity toward this receptor, compared to the A_1_ receptor (Table 1) [76]. In subsequent studies, the same group optimized 1,3,5-triazine scaffold and obtained, among others, compound **7c** (**2** in Figure 2), with improved affinity and selectivity for A_2A_ over the A_1_ receptor (Table 1) [77]. Other adenosine A_2A_ receptor antagonists in the group of [1,2,4]triazolo[5,1-*f* ]purin-2-ones were discovered by Basu et al. All of the synthesized compounds are characterized by high potency toward A_2A_ receptor and high selectivity over A_1_ adenosine receptors. The derivative 33 (3 in Figure 2) was further evaluated in behavioral tests with the use of haloperidol-induced catalepsy and 6-OHDA lesioned rat models, proving its efficacy after oral administration [78]. In contrast, Van Rensburg et al. suggest that combined adenosine A_2A_ and A_1_ receptor antagonism may be beneficial in alleviating symptoms of Parkinsonism, attributing improvement in cognitive deficits to blockade of A_1_ receptor. The group obtained 2-benzylidene-1-indanone derivatives as dual A_2A_ and A_1_ receptor antagonists. Compound **2c** (**4** in Figure 2) showed moderate affinities for these targets (Table 1) [79]. Continuing this concept, the same scientists designed and synthesized another series of benzocycloalkanone analogs by exchanging substituents in benzene rings. Among others, compound **2a** (**5** in Figure 2) with improved A_2A_/A_1_ receptor affinity ratio (Table 1) was reported [80]. An interesting approach consisting in dual-targeting of adenosine A_2A_ and dopamine D_2_ receptor was exploited by Shao et al., resulting in discovery of indolylpiperazinylpyrimidine derivatives as novel potential antiparkinsonian agents. Compound **5** (**6** in Figure 2) exhibits low micromolar affinity for the A_2A_ receptor (Table 1) and competitively replaces binding of (±)-2-(*N*-phenethyl-*N*-propyl)amino-5-hydroxytetralin hydrochloride ((±)-PPHT.HCl) with the dopamine D_2_ receptor. The compound was then evaluated in vivo in the *Drosophila* model of PD, showing improvement in motor functions and inhibiting the degeneration of dopaminergic neurons [81].

Although inhibitors of MAO-B, represented by selegiline and rasagiline, were approved for the treatment of PD years ago, currently, scientists are still highly focused on this molecular target. Especially, the outcomes of the ADAGIO (Attenuation of Disease Progression with Azilect Given Once-daily) study, indicating that rasagiline at a dose of 1 mg/day has disease-modifying effects in PD, attracted particular attention to this molecular target [82]. Kavully et al. proposed a new class of selective MAO-B inhibitors based on enamide (specifically cinnamamide) scaffold for potential treatment of PD. The more potent compound **AD3** (**7** in Figure 2) exhibited inhibition of MAO-B at low micromolar concentration (Table 1) and was proven to be a competitive, satisfactorily selective and reversible inhibitor of this enzyme [83]. Another team focused on monoamine oxidase B inhibitors developed novel compounds in the group of piperine derivatives with affinity for the target of interest. Among this series, esters (especially benzyl esters) were noticed to be more beneficial for activity at MAO-B than acids or amides, and α-cyano group was shown to lead to increased inhibition of this enzyme. In particular, compound **15** (**8** in Figure 2) showed a high inhibition rate at MAO-B and a high value of the MAO-B over the MAO-A selectivity index (Table 1). The competitive mechanism of MAO-B inhibition of piperine derivatives was indicated in kinetic studies [84]. The multi-target approach for drug design against PD was exploited by Tao et al., resulting in discovery of derivatives of coumarin Mannich base with combined activity against MAO-B and neuroinflammation. The most potent compound **24** (**9** in Figure 2) exhibited selective inhibition of MAO-B over MAO-A at a low micromolar concentration level (Table 1). Furthermore, the compound inhibits the release of nitric oxide in lipopolysaccharide-challenged BV2 cells, indicating its anti-inflammatory effect. Additionally, the studied compound displays some neuroprotective effect in L-glutamate and hydrogen peroxide models of cell injury. In in vivo studies, the compound was shown to significantly attenuate behavioral deficits associated with PD in the MPTP-induced mouse model, and protect tyrosine hydroxylase (TH)-positive dopaminergic neurons. Moreover, treatment with this compound suppresses the expression of cyclooxygenase-2 and inducible nitric oxide (NO) synthase (which are involved in generating inflammation) in the midbrains of MPTP mice [85]. Another series of compounds showing inhibition of MAO-B and possessing neuroprotective activity was published by Jismy et al. The group designed pyrimido[1,2-*b*]indazole derivatives, among which compound **4i** (**10** in Figure 2) displays inhibition of MAO-B in the nanomolar concentration range and high selectivity toward this target over MAO-A769 (Table 1). In the SH-SY5Y human neuroblastoma cell line, the compound exhibited protection against cell death induced by 6-hydroxydopamine [86]. Carradori and co-workers proposed dual-target inhibitors of MAO-B and acetylcholinesterase (AChE), based on thiazol-2-ylhydrazone scaffold. Compound **19** (**11** in Figure 2) is a selective, competitive and reversible inhibitor of MAO-B and also displays an inhibition of acetylcholinesterase (Table 1). Such dual-target profile may be beneficial for alleviating both motor and cognitive deficits in PD [87]. Affini et al. proposed affecting dopaminergic regulation by targeting both MAO-B and histamine H_3_ receptors. They designed a novel series of compounds by attaching a pharmacophore model of H_3_ receptor antagonist to indanone motif, which is one of the known scaffolds for MAO-B inhibitors. Compound **3f** (**12** in Figure 2) showed nanomolar MAO-B inhibition and moderate selectivity over MAO-A. This, combined with high affinity for H_3_ receptor (Table 1), makes the compound a promising lead structure for further optimization [88].

Current treatment of PD relies on alleviating its symptoms, thus there is still an unmet need to develop therapies that would modify the course of the disease. One of the potential therapeutic targets in this field is leucine-rich repeat kinase 2 (LRRK2). Increased activity of certain mutants of this kinase is believed to lead to damage of the dopaminergic neurons. Hence, attempts, however still limited, are made to develop LRRK2 inhibitors to target the cause of the disease. Osborne and co-workers discovered novel selective inhibitors of LRRK2 in the group of 5-azaindazoles. The compound **31** (**13** in Figure 2) is characterized by its potent ability to inhibit LRRK2 in low nanomolar concentrations (Table 1). It was deduced that the increase in potency and selectivity toward this kinase may be achieved by introduction of heterocycles in the position 3 of 5-azaindazole [89]. Indolinone scaffold was used by Salado et al. to design LRRK2 inhibitors as neurogenerative agents. The most potent compound **33** (**14** in Figure 2) exhibited inhibition of the kinase on a nanomolar level (Table 1). However, the blood–brain barrier permeability for this compound could not be predicted due to solubility issues. Other compounds from this series showed that LRRK2 inhibition promotes adult neurogenesis in neural stem cells (NSCs) isolated from the subventricular zone (SVZ) [90].

Another concept of suppressing the development of PD involves inhibiting of α-synuclein aggregation, which is one of the major events responsible for the progression of the disease. Although under physiological conditions α-synuclein plays a role in the regulation of neurotransmitters’ release, its overexpression and aggregation lead to toxic effects which are involved in the pathogenesis of PD, and thus suppression of these events is thought to be a potential strategy to manage the disease [91]. Maqbool et al. designed and synthesized a group of diphenyltriazine derivatives, among which some lower α-synuclein fibrillation by more than 50% and act as disaggregating agents in the in vitro thioflavin T (ThT) assay. Additionally, two of the obtained compounds, **A4** and **A8** (**15a** and **15b** in Figure 2), seem to ameliorate cytotoxicity induced by the aggregation of α-synuclein [92]. Another team reported derivatives of 4-aminopyridine as neuroprotective agents. From five synthesized analogs, compound **3** (**16** in Figure 2) displayed the most favorable properties. It inhibits α-synuclein expression by more than 50% compared to control, as determined by Western blot and quantitative densitometric analysis. It also shows some antioxidant and anti-inflammatory properties [93].

The targets described above are currently of the most interest among scientists searching for novel antiparkinsonian agents. Other reports from recent years concern inter-alia: anti-neuroinflammatory compounds targeting inducible NO synthase [94,95], cyclooxygenase-2 [95], histamine H_3_ and H_4_ receptors, agents affecting the Kelch-like ECH-associated protein 1 (Keap1)-nuclear factor erythroid 2-related factor 2 (Nrf2) signaling pathway [96,97], negative allosteric modulators of the GluN2B NMDA receptor [98], partial agonists of nociceptin opioid receptor (NOR) [99], modulators of β-Glucocerebrosidase [100], activators of UNC-51-like Kinase 1 (ULK1) [101] and phosphodiesterase-4 (PDE4) inhibitors [102].

## 6. Conclusions

Despite many studies on PD, there is still no effective treatment available. Although many pharmaceuticals are available, they are unfortunately only used for symptomatic treatment, which still makes the disease incurable and deadly. The current knowledge of molecular targets is still incomplete, but new reports on them are constantly being sought. For this reason, the most appropriate cell and animal models for PD studies are proposed to reflect the pathophysiological and behavioral aspects of the disease as much as possible. Moreover, the use by scientists of a new research tool—computer modeling—allows for more effective design of drugs. Therefore, thanks to advanced technology and the use of appropriate research models, it is possible to thoroughly understand the mechanisms of PD and to test new drugs on models that will reflect the disease entity in the human body.

## Figures and Tables

**Table 1:** Affinities and potencies of novel compounds at certain molecular targets (numeration according to Figure 2).

	*K*_i_ (nM)		EC_50_ (µM)	IC_50_ (µM)		% Inhibition at 3 µM	*K*_i_ (nM)	IC_50_ (nM)
	A_2A_R	A_1_R	D_2_R	MAO-B	MAO SI ^a^	AChE	H_3_R	LRRK2
1	32	322						
2	1.5	478						
3	1.5	1700						
4	903	435						
5	434	792						
6	11,200	>100,000	22.5					
7				0.11	>363			
8				0.047	>211			
9				3.66	>100			
10				0.13	>769			
11				0.0053	>501	44		
12				0.276	>36		6.5	
13								2
14								10

^a^ Selectivity index (SI) = IC_50_ MAO-A/IC_50_ MAO-B. *K*_i_—Dissociation constant; EC_50_—Half maximal effective concentration; IC_50_—Half maximal inhibitory concentration; AR—Adenosine receptor; DR—Dopamine receptor; MAO—Monoamine oxidase; AChE—Acetylcholinesterase; H3R—Histamine H_3_ receptor; LRRK2—Leucine-rich repeat kinase 2.

## Abbreviations

MPTP	1-methyl-4-phenyl-1,2,3,6-tetrahydropyridine
(±)-PPHT.HCl	(±)-2-(*N*-phenethyl-*N*-propyl)amino-5-hydroxytetralin hydrochloride
6-OHDA	6-hydroxydopamine
AChE	Acetylcholinesterase
AR	Adenosine receptor
AI	Artificial intelligence
BDNF	Brain-derived neurotrophic factor
cAMP	Cyclic adenosine monophosphate
*K*_i_	Dissociation constant
DR	Dopamine receptor
GDNF	Glial cell line-derived neurotrophic factor
EC_50_	Half maximal effective concentration
IC_50_	Half maximal inhibitory concentration
H3R	Histamine H_3_ receptor
iPSC	Induced pluripotent stem cells
LRRK2	Leucine-rich repeat kinase 2
MAO	Monoamine oxidase
MAO-B	Monoamine oxidase B
MAPT	Microtubule-associated protein tau
NGF	Nerve growth factor
NO	Nitric oxide
NOR	Nociceptin opioid receptor
NP	Network pharmacology
NSCs	Neural stem cells
NSE	Neuron-specific enolase
PD	Parkinson’s disease
PDE4	Phosphodiesterase-4
QSAR	Quantitative structure-activity relationship
SI	Selectivity index
SAR	Structure-activity relationship
SVZ	Subventricular zone
SN	Substantia nigra
SVM	Support vector machine models
SYN	Synpatophysin protein
THT	Thioflavin T
TH	Tyrosine hydroxylase
